# Strategic Framework for Increasing Accessibility and Utilization of Voluntary Counseling and Testing Services in Uganda

**DOI:** 10.1155/2011/912650

**Published:** 2011-06-30

**Authors:** E. Mugisha, G. H. van Rensburg, E. Potgieter

**Affiliations:** ^1^Program for Appropriate Technology in Health (PATH), P. O. Box 22616, Kampala, Uganda; ^2^University of South Africa, P. O. Box 392, Pretoria, South Africa

## Abstract

Despite the usefulness of VCT service as an entry point to prevention for the HIV-uninfected people and care, treatment and support for those who test HIV positive, VCT service remains poorly utilized among the fishing communities. The aim of the study was to identify factors influencing VCT service delivery and utilisation among fishing communities in Uganda and consequently, formulated a strategic framework for improving VCT service delivery and utilisation in the fishing communities. The study followed a 3-phased approach, collecting and analyzing quantitative data from Kasenyi fishing community under phase I, collecting and analyzing qualitative data from hospital managers and VCT counselors in phases II and III, respectively. Results indicate that VCT services delivery and utilisation is affected by factors at government (macro) level, the institution (meso) level, and at the individual (micro) level. Based on this, a strategic framework was designed, expected to increase VCT service availability, accessibility, and acceptability if applied. The researcher recommends the use of this useful tool in the design of VCT programs.

## 1. Introduction

HIV/AIDS in Uganda was detected as early as 1982 in Kashenshero, a small fishing village in Rakai District, which became the centre of the epidemic in the country, before spreading to the neighbouring fishing villages, and later to urban centres. In the 1990's, HIV prevalence rose to 18% before finally dropping to 6.0% in 2006 after behavioural change interventions [1]. Despite the gains made in reducing the HIV prevalence, there is concern that the presence of high HIV prevalence reservoirs in some fishing communities will erode the positive results [2]. Subsequently, the Ugandan government and its development partners have made serious efforts to ensure all Ugandans get voluntary counselling and testing (VCT) service whenever and wherever they want [3]. 

VCT is internationally recognized as an effective and important strategy for both HIV/AIDS prevention and care [4]. Furthermore, VCT has been found to be a cost-effective strategy for facilitating behaviour change [5]. VCT is, therefore, a core intervention in the comprehensive strategy of the government of Uganda and its development partners to address HIV/AIDS. Although several VCT sites have been set up in almost all health units across the country, VCT utilisation remains lower than projected [3]. Seeley and Allison [6] emphasise that poor VCT service utilisation makes it harder to deliver other AIDS-related care and treatment services since it is the only way of knowing one's HIV status.

### 1.1. Statement of the Problem

Several measures have been put in place to curb the spread of the deadly HIV virus, including the delivery of free or highly subsidized the quality VCT service. Despite the usefulness of VCT services as an entry point to prevention for uninfected people and care, treatment and support for those who test positive, the fishing communities in Uganda have not used this service as much as expected [2, 3].

### 1.2. Aim and Objectives of the Study

This study aimed at exploring and describing VCT service and factors that affect its delivery and utilisation in the fishing communities along the shores of Lake Victoria, in Wakiso District. The specific objective was to design a strategic framework to improve VCT service accessibility, acceptability, and utilisation among fishing communities.

## 2. Methods

The study was exploratory, descriptive, and explanatory and adopted a three-phased approach, collecting data from Kasenyi fish landing site residents in phase I, from VCT managers in phase II, and from VCT counsellors in phase III. Phase I collected quantitative data and phases II and III collected qualitative data (see Table 1). 

### 2.1. Quantitative Approach

Phase I used a quantitative approach in the sampling, collection, and analysis of data. The study population consisted of Kasenyi fish landing site residents. Kasenyi fish landing site is located in Katabi subcountry, Wakiso District, 25 kilometres from Kampala, the capital city of Uganda. The researcher selected 127 respondents (66 men and 61 women) by means of stratified random sampling. Data was collected through structured interviews and analysed using the SPSS (Version 12.0) program. The researcher took a positivist approach where he maintained a distance between himself as a research expert and the goings-on in the research settings. The researcher also took a position of an interested outsider [7].

#### 2.1.1. Validity and Reliability

The researcher ensured validity and reliability of the study instrument in several ways. Face validity was ensured through constructing questions relevant to the study aim and objectives; content validity was ensured through a comprehensive interview guide, using the literature reviewed. Besides, external validity was ensured through the use of random sampling techniques to select the final sample. 

Reliability of the study, which relates to the consistency of one's measurement, was also ensured [8]. The interview schedule was subjected to several pretesting and revisions which ensured that a reliable tool was used to collect the data. Besides, experts well versed with VCT service were asked to review and advise on the interview schedule.

### 2.2. Qualitative Approach

Phases II and III were qualitative in nature, the phase II population consisted of VCT managers, who were selected from two hospitals in Wakiso District. One VCT manager at each of the hospitals was interviewed as a case study and qualitative data collected. 

Phase III population consisted of VCT counsellors at each of the hospitals. The researcher purposively selected 7 VCT counsellors at the hospitals, collected, analysed, and interpreted qualitative data, therefrom. VCT counsellors who had worked at the hospital less than three years were excluded from the study.

#### 2.2.1. Trustworthiness

Trustworthiness in the qualitative approach was achieved through three main ways, namely, dependability, confirmability, and credibility [9]. Dependability which includes activities that increase the credibility of findings was ensured through logging all sessions dealing with collection and interpretation of data and keeping track of how coding evolved. Confirmability which relates to the objectivity or neutrality of the data was ensured through both the researcher and the research-assistant generating field notes and interview transcripts, which were used during analysis. Besides, at the end of each interview, the researcher reconfirmed the main points with the participant. Lastly, credibility which relates to confidence about truth of the data was achieved through vigorous training of the research assistant.

### 2.3. Theoretical Approach

The interplay between health services delivery and health services utilisation can be explained at macro, meso, and microlevels, using a framework developed by Aday and Andersen in the 1970s [10], and this was the basis of the formation of the strategic framework for improving VCT services. 

At the macrolevel, the researcher analysed the large-scale social systems involved in VCT delivery such as government policies, guidelines, and resource allocation [10]. Based on the findings from phase I, macro level factors that seemingly have an effect on VCT service delivery and utilisation were identified and included in the strategic framework design.

At the mesolevel, the focuse was on organisations' (health facilities), factor that affects VCT service delivery and utilisation. Considered here is the health delivery system, which is the arrangements for the potential rendering of care to consumers, which are mainly influenced by the available resources and the structure of the organisation [11]. Results from both phase I and II were used for this level of analysis. 

At the microlevel, two main elements were considered, namely, the characteristics of the target population at risk and consumer satisfaction. The characteristics of the population at risk include predisposing, enabling, and need components, which are the individual determinants of health service utilisation. The predisposing factors include variables that describe the tendency of individuals to use or not to use the services. Results from phase I helped inform this level.

### 2.4. Experts' Review of the Strategies

Once the strategies for increasing VCT accessibility and acceptability were drafted as described above, they were presented to a selected team of experts in different fields for a purposive review. This was done in order to assess whether the strategies could be acceptable as described, acceptable but with recommendation, or not acceptable at all. The convergent process was used which includes consensus formation regarding issues that need to be addressed, their priority, level and the most efficacious means to address them [12].

Five experts in the areas of academics, HIV/AIDS, community, policy, and counselling were purposively selected to critique the strategies. The experts were asked to read a summary of the research findings, review the draft strategies, and give their comments on the assessment form. A matrix form based on the Likert scale was provided to the experts in order to rate the strategies [13]. The responses were analyzed and fed into the design of the strategic framework for VCT service delivery and utilization.

## 3. Results

The factors that affect VCT service accessibility, acceptability and utilisation are summarised according to the three levels in the health services delivery and utilisation framework, namely, macro, meso, and microlevel.

### 3.1. Macrolevel

The macrolevel involved analysis of the large-scale social systems involved in VCT the delivery such as government policies and guidelines. In terms of resource allocation, funding for VCT service is largely determined at the macrolevel. Accordingly, at macrolevel, access to health services has been considered mainly in a political context, where governments have the power and mandate to direct healthcare systems through health policy [10, 14].

#### 3.1.1. Inadequate VCT Guidelines

Although VCT service utilisation trends are changing, they are not equally matched by policy adjustments, and this is likely to hamper service delivery. The VCT managers in phase II held that current VCT policy guidelines were not adequate for VCT service delivery at the present time. The VCT counsellors in phase III felt that the guidelines were not applicable to all the situations they dealt with. The VCT counsellors cited the example of minors being required to have a parent's or guardian's consent in order to be tested for HIV, which could be difficult.

#### 3.1.2. Poor Referral Systems

 Comprehensive service delivery appeared to work well in an integrated system, with opportunities for making internal referrals. However, poor followup of external referrals could reduce client satisfaction and, hence, utilisation. According to the VCT managers in phase II, there was no effective external referral system for cases which the hospitals could not handle, and even when referrals were made; there was no followup to know that the referrals had actually happened.

### 3.2. Mesolevel

The mesolevel focuses on organisations, for example, health facilities that provide VCT services. It considers the health delivery system as those arrangements for the potential rendering of care to consumers, which mainly involve the available resources and the structure of the organisation [11].

#### 3.2.1. VCT Service Delivery Models

 VCT service is generally available onsite or offsite through mobile VCT. There is an ever-increasing demand for VCT, therefore, the participants were of the opinion that such an increase should be matched with increased staffing levels and appropriate policy guidelines. Although the integration of VCT services with related services is good, it has put a strain on other services, because it has not been matched with funding and staffing demands. While VCT counsellors preferred mobile VCT service, it suffers from transport problems and demand for services that exceeds what the counsellors can offer.

#### 3.2.2. Limited Staff for VCT Services

 The study found a shortage of VCT counsellors at the hospitals. In addition, the few who were available were required to offer a wide range of other health services in the same environment. This could affect the quality of VCT services provided and, hence, a barrier to accessing VCT services. For example, the VCT managers in phase II indicated that counsellors were sometimes called for emergencies while counselling clients and, therefore, had to temporarily abandon VCT clients. Both the VCT managers (phase II) and VCT counsellors (phase III) suggested that community volunteers be identified and trained to offer counselling.

#### 3.2.3. Limited Counsellor Support

 The findings indicated that counsellor support was almost nonexistent at the research sites, which can lead to low quality of VCT service, and sometime counsellor burn out. The VCT managers in phase II had no clear means of measuring counsellors' performance or offering them support supervision. To them, this was complicated by the nature of integrated service delivery. The VCT counsellors in phase III stated that although staff meetings took place, they reviewed general issues, not necessarily VCT-related issues in particular. Furthermore, supervisors emphasised numbers tested rather than considering the quality of counselling offered.

#### 3.2.4. Motivation to Offer VCT Services

 The VCT counsellors in phase III at both hospitals were highly motivated to offer VCT services, and many indicated they had not been stressed despite the existence of stressors. Many of the counsellors in phase III indicated that they enjoyed their work. Among the respondents in phase I who had accessed VCT services, there was a general feeling of satisfaction with the way VCT services were delivered, apart from complaints about the long time spent waiting at the hospitals.

#### 3.2.5. Simple HIV Testing Methods

 Same-day, single visit VCT is one of the ways of ensuring that most clients who test receive their test results, as they did not have to return another day. Moreover, individuals who need to make immediate decisions based on HIV test results find single visit testing more convenient. The VCT counsellors in phase III felt that rapid testing had assisted many people to access their results and helped to save resources for both the service providers and service users. The respondents in phase I who had tested for HIV had received their HIV test results because they had tested and received results the same day during the same visit.

### 3.3. Microlevel

The microlevel considers two main elements, namely, the characteristics of the target population at risk and consumer satisfaction. The characteristics of the population at risk include predisposing, enabling and need components as the individual determinants of health service utilisation. The predisposing factors include variables that describe the tendency of individuals to use or not to use the services. Consumer satisfaction refers to the attitudes towards the medical care system of those who have experienced contact with it [10, 15].

#### 3.3.1. Limited Knowledge and Awareness about VCT Services

 Although participants were generally well aware of HIV/AIDS, its spread and prevention, there was limited knowledge of the benefits of testing for HIV. While the respondents in phase I knew about HIV acquisition and prevention, a quarter of the respondents still believed it was possible to tell people's HIV status just by looking at signs and symptoms. This possibly accounted for the majority of the Kasenyi residents attending VCT centres when they found signs and symptoms presumably associated with HIV/AIDS. In addition, the VCT managers in phase II pointed out that many clients who accessed VCT services believed that they were infected with HIV and were only looking for ART services.

#### 3.3.2. Fear of HIV Test Results

 Clients' fear of test results was one of the major challenges in counselling. The fear associated with self-diagnosis of HIV based on mere signs and symptoms presumably related to HIV creates anxiety, which could be a hindrance to accessing HIV testing. Fear of the test results was one of the major barriers to testing raised by the respondents in phase I. According to the VCT counsellors in phase III, clients' anxiety was a major challenge, which interfered with the delivery of key messages. This anxiety was due to the anticipation of HIV-positive results. Likewise, when asked about the reasons for testing (among those respondents who had an HIV test), most men said that they tested after getting signs and symptoms possibly related to HIV/AIDS. 

#### 3.3.3. Community Attitudes Towards VCT Service

 According to phase I findings, stigma related to HIV testing appeared to be lessening in communities and discussing HIV/AIDS-related issues with community members could have a positive impact on taking an HIV test. The findings indicated that of the respondents in phase I, 97.6% agreed that HIV testing was a good thing and 94.5% were willing to refer others for testing. Furthermore, 60% of the respondents in phase I indicated that they had no problems with their HIV test results being known in their communities.

#### 3.3.4. Problem of Waiting Time

The respondents in phase I complained of long waiting hours, the VCT counsellors in phase III did not agree that the waiting time was always long despite emergency situations that might arise. Instead, they reported that clients accessing VCT services experienced abnormally high anxiety, which to clients made the waiting time seem much longer. In addition, the VCT managers in phase II stated that VCT clients always wanted to be served as soon as they arrived and as quickly as possible.

#### 3.3.5. Willingness to Test for HIV

Many of the respondents in phase I, both who had and who had not tested before, indicated that they were willing to test and would find it easy to test. Moreover, most of the respondents agreed that HIV testing was a good thing and were willing to refer others for testing. Under favourable conditions, therefore, individuals would be encouraged to consider testing for HIV.

### 3.4. Strategic Framework for VCT Services

The findings of the study summarised above enabled the researcher to devise strategies for increasing VCT accessibility, acceptability, and utilisation in fishing communities. The strategies later fed into the design of the strategic framework (Table 2) which is based on study findings that VCT providers at hospitals are willing to increase the quantity and quality of VCT services to the target communities, and the respondents from the target community in phase I indicated willingness to access VCT services. 

The strategic framework includes six key strategic activities for increasing VCT accessibility, acceptability and utilisation. These are the following: improving advocacy for VCT among leaders, increasing awareness and mobilising communities, promoting provider-initiated HIV counselling and testing, creating a favourable and enabling environment for clients seeking VCT, exploring other VCT staffing alternatives, and progressively monitoring of VCT services (see Table 2 ). 

In Table 2, each of the six strategies (column 1) is justified (column 2), and key findings from the study that support the strategy are presented (column 3), and lastly recommendations for service delivery (column 4).

## 4. Discussion

VCT campaigns are a critical step in improving VCT service. Hospitals should conduct campaigns to educate people at all levels about the benefits of VCT services. According to Harrington and Harrigan [16], it is the responsibility of practitioners to take a leadership role in advocacy to promote counselling and testing. One of the expert reviewers indicated that health workers lacked advocacy skills. Therefore, in order to reap the benefits of advocacy, health workers need to be trained in the art of advocacy.

In Uganda, mass media and marketing approaches have proved successful in improving people's perceptions of the benefits of knowing their status and increasing the uptake of VCT in some communities [17]. VCT staff, especially counsellors who have had interactions with clients, should be involved in designing communication messages and promotional activities. The use of the radio would be more appropriate to this population, where 83.5% of the respondents in phase I indicated they commonly listened to the radio almost daily. In Nigeria, Falobi et al. [18] found a huge potential for mobilising masses against AIDS through the radio.

According to the FHI [19], effective communication for increasing demand for VCT services may include offering information on where VCT service is available, including the availability of related HIV/AIDS services; addressing the benefits of HIV testing; encouraging target populations to access and utilise VCT services; encouraging sustained behaviour change after a person has been tested, and encouraging counselling and testing as a routine component of health-seeking behaviour. 

When approached for VCT services, clients have the option to “optin” or “optout”. “Optin” generally refers to counselling and testing where a client explicitly consents to the test, whereas “optout” allows individuals to specifically decline the HIV test having received pretest information. Nevertheless, regardless of whether they test right away, the information received is likely to influence them to test in the future. Grover and Petterson [20] found that of 364 women offered HIV screening during a six-month period, 248 (68%) accepted and underwent testing. 

The use of community volunteer counsellors could be an additional resource to the inadequate staff. Their selection, however, should be done with great care to avoid negative outcomes and ensure that they are accepted in their communities. In their study, Kipp et al. [21] found that some study participants recommended that counsellors be nonresidents of the area, as nonresidents were considered more credible and would offer greater confidentiality than residents. 

Lastly, VCT service should be closely monitored and evaluated with an aim of making improvements. According to the FHI [22], monitoring and evaluation is a critical component of VCT service delivery and should address two relevant areas for service providers and policy makers. For service providers, it should examine how well VCT service is provided while for policy makers, it should determine programme effectiveness, and the impact of VCT on the population receiving the service.

## 5. Conclusion

In the communities that have been longest and hardest hit by the HIV/AIDS epidemic, an increasing number of people need to have access to prevention, care, treatment, and support services. This, however, can only be possible if the individuals have access and fully utilise VCT services. Accessibility, acceptability, and utilisation of VCT services is largely dependent on three main factors, namely, government involvement in VCT services (macrolevel), service provider characteristics (mesolevel), with regard to the model of providing the VCT services and the characteristics of the target community (microlevel), which considers their knowledge, attitudes, and perceptions towards VCT services. Understanding the interplay of these three levels and how they can better be influenced will go along way in increasing VCT service delivery and utilisation. The VCT service strategic framework is a tool expected to increase VCT accessibility, acceptability, and utilization among fishing communities.

## Figures and Tables

**Table 1:** Phases of the study.

	Phase I	Phase II	Phase III
Approach	Quantitative	Qualitative	Qualitative
Population	Kasenyi fishing community	VCT managers at the two hospitals in Wakiso District	VCT counsellors at the two hospitals in Wakiso District
Sampling method	Stratified random sampling	Purposive (whole population)	Convenient sampling
Sample	127 members of the Kasenyi fishing community (61 women and 66 men)	2 VCT managers	7 VCT counsellors
Instrument	Structured interview	Interview guide	Interview guide
Data analysis	EPI data version 3 and SPSS version 12.0	Word processor	Word processor

**Table 2:** Strategic framework for improving VCT accessibility and acceptability.

Strategy	Justification	Key findings that support the strategy	Recommendations for service delivery (practice)
(1) Improve advocacy for VCT among leaders	(i) Advocacy is likely to increase the volume and quality of VCT services (ii) Involvement of local leaders in VCT programmes is important (iii) Advocacy of relevant key stakeholders is likely to generate support and funding for VCT services	(i) VCT managers indicated limited resources for VCT services and yet they believe if VCT are prioritised, there is likely to be financial support (ii) Respondents indicated the significant role their local leader could play in increasing VCT accessibility (iii) VCT managers indicated that sometimes there is poor response in community VCT due to poor mobilisation	(i) There is a need to understand the appropriate medium of communication for different stakeholders (ii) Hospitals should conduct promotional campaigns for counselling and testing aimed at raising support for VCT services (iii) Strong advocacy and synergising strategic approaches and efforts of various sectors, including the media, should be designed and implemented (iv) Health workers need to be trained in advocacy skills
(2) Increase awareness and mobilise communities	(i) Awareness of VCT services and the benefits of testing is a major factor in accessing and using the services (ii) When communities are mobilised, it enables the members to make informed decisions about VCT utilisation (iii) Talking freely about HIV AIDS reduces stigma and increases chances of testing	(i) Among the Kasenyi respondents, 23.6% thought that people could tell HIV status without necessarily testing (ii) Findings from respondents in phase I indicate that 27.6% are not aware of VCT services (iii) 11% did not know where to access VCT services (iv) If well mobilised, 89.8% of the respondents said it would be easy for them to go for an HIV test (v) VCT managers indicated that advertising for VCT services is limited, relying only on word of mouth (vi) Radio is listened to by 83.5% of participants at Kasenyi, thus good media (vii) 56.7% indicated fear of HIV results as a major barrier to testing (viii) 60% of respondents had not heard any message about VCT in the last 3 months (ix) Discussing issues related to HIV was found to be associated with HIV testing, as 96.7% of respondents who had tested had discussed HIV issues with someone	(i) Communities should continuously be given enough information so as to see VCT as a norm, which will reduce fear for HIV testing, reduce stigma, and increase the uptake of the services (ii) The use of radio in transmitting VCT messages would be the best medium of communication for the Kasenyi fish landing community (iii) It is important to encourage community members to talk about HIV in general and VCT in particular as it is likely to lead to testing (iv) Involve individuals in the target communities in the planning and implementation of VCT services
(3) Promote provider- initiated HIV counselling and testing	(i) It is a basic responsibility of health care providers to recommend HIV testing and counselling as part of routine clinical management (ii) Patients attending hospitals for health services could decide to take a test if health providers inform them about it	(i) According to the VCT managers, mothers coming for antenatal services are told of VCT services, and the majority accept and they are tested (ii) Findings from the Kasenyi respondents indicated that 25% of those who had tested had been asked to, but were happy to have tested (iii) VCT managers indicated that provider-initiated HIV testing and counselling is recommended as long as it is voluntary and follows principles of informed consent, counselling and confidentiality	(i) Whenever individuals attend a health centre, whether seeking health services or escorting a patient, they should be informed of the availability of VCT services and the importance of testing for HIV (ii) Whether an HIV test is requested by a health provider or not, pretest counselling should always be provided and confidentiality ensured
(4) Create an environment conducive to clients seeking VCT services	(i) Individuals are more likely to demand VCT services on their own if there is an enabling, favourable environment (ii) If potential clients are assured of privacy and confidentiality, the utilisation of VCT services is likely to increase	(i) There is a positive attitude towards testing. Of the Kasenyi respondents, 97.6% indicated that VCT is a good service (ii) There is a need to create a favourable environment. For example, some of the Kasenyi respondents emphasised the need for privacy and confidentiality in testing (iii) At testing sites, VCT counsellors indicated their efforts to making VCT service responsive to client and community needs and priorities	(i) There is a need to improve other health services as well, not just VCT services (ii) Mobile VCT services should be considered for special populations such as people in remote rural areas and without access to health services, so as to make it easy for them to access VCT services (iii) Assurances of privacy and confidentiality as well as trustworthiness are key factors in individuals' decision to test (iv) Testing should be voluntary and routinely offered to clients rather than clients having to request it
(5) Explore other VCT staffing alternatives	(i) Lack of adequate and well-trained VCT personnel is a major challenge in health services delivery (ii) VCT clients do not like spending unnecessary time at VCT sites	(i) VCT managers and counsellors indicated that the integration of VCT services with other services without additional staff has created staff shortages (ii) VCT staffing shortages were evident, when VCT counsellors and managers indicated that they prioritise emergencies, and VCT is not an emergency (iii) VCT counsellors indicated that community members could be trained to at least offer precounselling services in their communities (iv) The availability of averagely educated residents at Kasenyi could be a potential for working as community volunteer counsellors (v) Limited staff means more time clients spend at VCT sites. For example, 30% of respondents did not like the long time they spent at VCT sites	(i) With limited funding, use of community volunteers could be considered as an option (ii) Community volunteer counsellors could be a cheaper alternative source for the much needed human resources (iii) Great care needs to be taken while selecting community volunteers to ensure that they will be accepted in their communities
(6) Progressive monitoring of VCT services	(i) The best way to understand and improve the quality of VCT is to continuously assess the services (ii) Collection of essential basic data is one of the ways of assessing the performance of VCT services (iii) When funds are limited, as in this case, monitoring does not need to be massive and expensive, but just collection of basic data	(i) Collection of basic statistics on VCT services utilisation was not done at VCT sites (ii) Client satisfaction assessments as a means of improving VCT quality are not often done (iii) Some respondents who had accessed VCT services were not happy with certain elements of VCT services, such as too much time spent at the site (iv) Counsellor support supervision was noted to be missing at the VCT sites	(i) Support supervision and giving immediate feedback to VCT counsellors is of great value (ii) Periodic client satisfaction should be evaluated to match the needs and requirements of the VCT clients (iii) Counsellors should be interested in learning new counselling skills, be comfortable in discussing specific-HIV risk behaviours, and receive periodic support supervision (iv) Simple methods of monitoring VCT service delivery such as exit interviews or suggestion boxes could be used
